# Performance Characteristics of Anti–Collagen II Antibodies in Relapsing Polychondritis and Related Diseases: Prospective Analysis, Systematic Review, and Meta‐Analysis

**DOI:** 10.1002/acr.25697

**Published:** 2026-02-11

**Authors:** Karyssa Stonick, Marcela A. Ferrada, Alice Fike, Kaitlin A. Quinn, Benjamin A. Turturice, Casey Stein, Peter C. Grayson

**Affiliations:** ^1^ National Institutes of Health Bethesda Maryland; ^2^ University of Maryland Baltimore

## Abstract

**Objective:**

Relapsing polychondritis (RP) is a rare disease defined by recurrent cartilaginous inflammation. Anti–collagen II (Col2) antibodies have been proposed as a diagnostic biomarker for RP, but their performance characteristics are not well defined.

**Methods:**

In an observational cohort, anti‐Col2 antibody levels were measured in patients with RP compared to other inflammatory diseases that mimic RP. In parallel, a systematic review and meta‐analysis was performed to assess anti‐Col2 antibody prevalence across a broad spectrum of diseases. Individual study risk ratios (RRs) and pooled disease category RRs, study bias, and interstudy heterogeneity were assessed.

**Results:**

In the observational cohort, anti‐Col2 antibody prevalence did not differ between RP and comparators. The performance characteristics of anti‐Col2 to diagnose RP were poor (sensitivity = 18%; specificity = 72%). Anti‐Col2 antibody titers did not correlate with disease activity in RP (*r* = 0.08, *P* = 0.44). In the systematic review, 71 of 2,443 reviewed articles were included. Anti‐Col2 antibodies were not associated with RP across five pooled studies (RR: 2.09; 95% confidence interval [CI]: 0.05–81.80; *P* = 0.69). Anti‐Col2 antibodies were significantly associated with a composite group of inflammatory diseases with cartilaginous involvement (RR: 2.99; 95% CI: 1.29–6.91; *P* = 0.01). Studies using healthy controls reported increased effect sizes compared to studies that used disease controls (β‐estimate = 1.14, *I* ^*2*^ = 17.08%; *P* = 0.0004).

**Conclusion:**

Anti‐Col2 antibodies are neither sensitive nor specific for RP, are detected in the minority of patients with RP, and are detected at similar prevalences across a spectrum of inflammatory diseases with cartilage inflammation. Use of these antibodies to diagnose or monitor RP is not advisable.

## INTRODUCTION

Relapsing polychondritis (RP) is a rare, systemic inflammatory disease characterized by recurrent episodes of cartilage inflammation that can lead to organ and life‐threatening complications. RP is primarily diagnosed based on clinical recognition of an appropriate pattern of symptoms and organ system involvement. The clinical criteria to diagnose RP include auricular, nasal, and airway/laryngotracheal chondritis, seronegative nonerosive arthritis, ocular inflammation, hearing loss, vestibular damage, response to glucocorticoids or dapsone, and histologic confirmation.^1^, ^2^, ^3^ Given the rarity and heterogeneous nature of RP, developing adequate expertise to diagnose the disease is challenging, which can lead to diagnostic delays.^1^, ^2^, ^3^ Identification of biomarkers that can accurately and objectively confirm disease when there is clinical suspicion remains a major unmet need in RP.

**SIGNIFICANCE & INNOVATIONS**

- The performance characteristics of anti–collagen II autoantibodies to diagnose or monitor relapsing polychondritis (RP) are poor.
- Presence of anti–collagen II antibodies likely indicates a history of cartilage inflammation rather a disease‐specific pathologic antibody.
- Anti–collagen II antibodies have limited utility as a clinical biomarker and should not be routinely ordered in cases of suspected RP.

Anti–collagen II (Col2) antibodies have been associated with RP, and testing for these antibodies is clinically available.^4^, ^5^, ^6^, ^7^ A few small studies have reported an association between anti‐Col2 antibody titers and RP.^4^, ^5^, ^6^ Animal models of disease support a potentially pathogenic role of anti‐Col2 antibodies in RP and related diseases. Immunization of mice with anti‐Col2 antibodies induces collagen‐induced arthritis and ear chondritis, which recapitulates clinical features of both rheumatoid arthritis (RA) and RP.^8^, ^9^ Anti‐Col2 antibodies have been proposed as a potential diagnostic biomarker for RP. Whether these antibodies are reliable clinical biomarker to diagnosis and monitor RP has not been well characterized, and it remains unclear if anti‐Col2 antibodies are directly involved in disease pathogenesis in humans or simply represent a marker of cartilage disease.

The objective of this study was to determine the performance characteristics of anti‐Col2 antibodies to differentiate RP from other diseases. Performance of anti‐Col2 antibodies was assessed within an observational cohort study of patients with RP and comparator diseases that can present with similar cartilaginous and extracartilaginous features as RP. A systematic review and meta‐analysis was also performed to synthesize existing evidence on the prevalence and diagnostic utility of anti‐Col2 antibodies in RP and other conditions.

## METHODS

### Cohort study

#### Study population

Patients with RP and inflammatory diseases with clinical overlap were enrolled into an ongoing, prospective observational cohort study at the National Institute of Arthritis, Musculoskeletal, and Skin Diseases of the National Institutes of Health. Each patient provided written informed consent, and the protocol was approved by local ethics review (14‐AR‐0200). All patients with RP fulfilled diagnostic criteria for this disease.^1^, ^2^, ^3^ A subset of patients with RP who have mouth and genital ulcers with inflammatory chondritis (MAGIC) syndrome were studied independently. A comparator group of patients with other forms of vasculitis was selected from within the same study protocol, including patients with vacuoles, E1 enzyme, X‐linked, autoinflammatory, somatic (VEXAS) syndrome, large vessel vasculitis (LVV), or small and medium vessel vasculitis (SMVV). Patients with LVV included those with giant cell arteritis or Takayasu arteritis. Patients with SMVV included those with granulomatosis with polyangiitis (GPA), Behçet disease, microscopic polyangiitis, eosinophilic GPA, isolated cutaneous vasculitis, cryoglobulinemic vasculitis, or IgA‐associated vasculitis. The composite disease comparator group was chosen because these conditions have clinical features that can substantially overlap with RP, and thus are a likely population in which a diagnostic test to rule out RP would be clinically indicated.

#### Clinical assessment

Study investigators clinically evaluated each patient at every study visit per protocol. Clinical features of disease were systematically recorded. Chondritis was defined as auricular chondritis, nasal chondritis, airway chondritis, or inflammatory arthritis. Cartilage damage was defined as subglottic stenosis, tracheomalacia, bronchomalacia, saddle nose deformity, or external ear deformity, as previously defined.^10^ Physician Global Assessment (PGA) scores were assessed on a scale of 0: remission to 10: severe disease at each visit. Demographic information including age, sex, race, ethnicity, CD19 B cell counts, and treatments was recorded. Patients were considered B cell depleted if they had an absolute CD19 cell count of <70/uL by flow cytometry.

#### Anti‐Col2 antibody testing

Anti‐Col2 antibody testing (Mayo Clinic, test identifier: FFTYC) was performed on the serum of consecutive patients evaluated between 2019 and 2023. If testing from multiple study visits was available for an individual patient, results from the initial test was used. The Mayo Clinic test uses an IgG antibody against the antigen of denatured Col2 derived from chick sternal cartilage. Reference ranges were applied per predefined test standards from the Mayo Clinic: an anti‐Col2 antibody titer level of 20 to 25 equivalent units (EU)/mL was considered borderline elevated, and a titer level of >25 EU/mL was considered unequivocally positive. Analyses were performed at both cutoff levels.

#### Statistical analysis

Performance characteristics of anti‐Col2 antibodies (sensitivity, specificity, receiver operating characteristic curves) were assessed in patients with RP and MAGIC syndrome in reference to the comparator disease group (R‐Studio pROC).^11^ The comparator group was further stratified into diseases of cartilaginous inflammation (VEXAS, GPA) and extracartilaginous symptom overlap (all other comparators). Continuous variables were compared between groups using Kruskal‐Wallis tests. Correlations were calculated using Spearman rank coefficient. Categorical variables were compared using Fischer's exact tests or chi‐squared test, as appropriate. The frequency of specific clinical symptoms was compared between those who were anti‐Col2 antibody positive vs antibody negative for patients with RP alone and for patients with diseases of cartilage inflammation (RP, MAGIC, VEXAS, GPA). Analyses were performed using GraphPad Prism 10, JMP 16, and R‐Studio 4.4.0.

### Systematic review and meta‐analysis

#### Literature search

To evaluate the diagnostic performance of anti‐Col2 antibodies across a broader spectrum of diseases, a systematic review and synthesis of evidence was performed in accordance with the recommendations of the Cochrane Collaboration and the Preferred Reporting Items for Systematic Reviews and Meta‐Analyses (PRISMA). The study was registered in PROSPERO (April 1, 2025; Centre for Reviews and Dissemination no. 1023286). A comprehensive PubMed search was performed using the terms ((anti‐collagen II) OR (collagen II antibodies)) AND (human)), and snowballing was performed to identify any additional articles to include. One study investigator (KS) performed the article search. Rayyan AI was used to increase the efficiency of the review by highlighting words that the authors designated as keywords for inclusion (i.e., human, collagen II, etc.) and exclusion (i.e., animal, mouse, rat, etc.) and by tracking which articles were marked for inclusion/exclusion.^12^ Articles were excluded first by title, then abstract, and finally by full article review. Studies were excluded if they (1) only measured anti‐Col2 antibody titers in animal models, (2) did not include the data from the control population and/or only used the control population to set the reference range within their study and did not show the rate positivity of anti‐Col2 antibodies within the control population, (3) did not report the rate of positivity or number of participants positive within the study/disease population, (4) did not measure anti‐Col2 antibodies in serum (ie, only measured it in synovial fluid), (5) were not printed in English, or (6) were unable to be accessed. Studies were included in the meta‐analysis if they were published in English, were accessible, and measured and reported anti‐Col2 antibody prevalence rates in the serum from at least one human disease/participant population and the serum from at least one human control population. Positivity definitions from each study were used for that respective study to define the rate of positivity in the disease/participant population and control population. Individual study definitions for anti‐Col2 positivity were frequently based on the titer levels of the control population (ie, ±2 or 3 SDs from the average titer level of the control population).

#### Study data extraction

For each study, one study investigator (KS) extracted and recorded the sample size, proportion of participants who were anti‐Col2 antibody positive, proportion of controls who were anti‐Col2 antibody positive, whether healthy and/or disease controls were used, year of publication, country of publication, proportion of female participants (if reported), Col2 antigen species (if reported), Col2 antigen anatomic location (if reported), whether native or denatured anti‐Col2 antibodies were used (if reported), average age of the participants (if reported), and the quality of the study. The quality of each study was assessed using the Newcastle‐Ottawa Quality Assessment Scale for Case Control Studies. For comparability, studies should account for treatment effect for therapies that could impact antibody production. Additionally, composition of the control group (healthy vs appropriate disease comparator conditions) was considered when assessing study quality.

If more than one disease/population was investigated within an article, the disease that was emphasized in the discussion and conclusion was designated as the primary disease for that study. To reduce multiple comparisons, only the primary disease and control populations were included in the meta‐analysis. If multiple antibody comparisons were included, the one most similar to the antibody used by the Mayo Clinic test was included (IgG, denatured, chick, sternal cartilage). If multiple control populations were included, the comparison to the disease control population rather than the healthy control population was included.

#### Study categorization

Studies were grouped into four categories based on classification of the primary disease of interest as predisease, noninflammatory diseases, inflammatory diseases without cartilage involvement, and inflammatory diseases with cartilage involvement (see Supplementary Table 1 for definitions). Two authors (CS, BAT) independently categorized all the articles included in the study according to these definitions. Discrepancies were resolved with review by a third author (KAQ) and discussion.

#### Statistical analysis

Risk ratios (RRs) and 95% confidence intervals (CIs) were calculated for each study, and pooled RRs were estimated using the restricted maximum likelihood method. Studies were weighted by cohort size relative to the total number of participants in all the studies within that category combined (equation: (no. of participants within the disease cohort of an article) / (sum of the number of participants with the disease cohort of all the studies) = weight of the study). This method was chosen rather than inverse variance to minimize weighting of studies where the total sample size was disproportionally driven by controls rather than cases. Study bias was calculated using Egger's test. Heterogeneity between studies was evaluated using meta‐regression analyses. Forest plots, funnels plots, and meta‐regression plots were generated to visualize the data. For meta‐regression analyses, the following variables were considered: publication year, sample size of the disease cohort, total study size, age and sex of patients in the disease cohort, disease category (predisease, noninflammatory, inflammatory with or without cartilage involvement), country of publication (Americas, Australia and Asia, Europe), antigen species (human, nonhuman, both), antigen location (axial, appendicular), and control population (healthy vs disease). Studies with missing data or that did not fit into the defined subcategories above were excluded. Subcategories with a minimum of two studies were included in the final regression analysis. Variables with a *P* value < 0.20 and an *R* ^2^ > 0% on univariable regression were included in multivariable models (R‐Studio metafor, ggplot2).^13^, ^14^ R‐Studio (meta, metafor) was used to run meta‐analysis and meta‐regressions.^11^, ^14^, ^15^

## RESULTS

### Cohort study

#### 1. Patient characteristics

A total of 171 patients were included in the present study: 84 with RP, 11 with MAGIC, and 76 with comparator diseases (45 with VEXAS, 13 with LVV, and 18 with SMVV). Baseline demographics including sex, race, and factors relevant to treatment and disease, reported by disease category, are displayed in Table 1. Except for the patients with VEXAS, which consisted entirely of men because it is an X‐linked disease, the cohort was predominantly female (Table 1). A proportion of patients within each disease category were B cell depleted at the time of antibody testing (Table 1)—this was sometimes, but not always, due to B cell–depleting therapy. Median PGA scores significantly differed by disease (*P* < 0.0001; Table 1), which likely reflects lack of effective clinical treatments for RP, MAGIC, and VEXAS relative to other forms of vasculitis.

**Table 1:** Study population characteristics for the observational cohort (n = 177)*

Characteristics	Relapsing polychondritis (n = 84)	MAGIC (n = 11)	VEXAS (n = 45)	LVV (n = 13)	SMVV (n = 18)
Age, median (IQR), y	45.5 (33.0–57.25)	41.0 (26.5–48.5)	67.0 (63.0–72.0)	37.0 (23.0–69.0)	62.5 (39.5–69.25)
Female sex, n (%)	60 (71)	10 (91)	0 (0)	12 (92)	13 (72)
Race, n (%)	74 (88)	10 (91)	42 (93)	11 (85)	15 (83)
CD19 absolute count <70/uL, n (%)	2 (2)	3 (27)	33 (73)	3 (23)	8 (44)
B cell–depleting therapy, n (%)	3 (4)	0 (0)	4 (9)	0 (0)	4 (22)
IVIG, number (%)	7 (8)	1 (9)	0 (0)	0 (0)	1 (6)
Prednisone dose, median (IQR)	3.75 (0.00–12.12)	0.00 (0.00–15.00)	20.00 (10.00–25.00)	0.00 (0.00–4.00)	0.00 (0.00–5.00)
PGA scores, median (IQR)	2.00 (1.00–3.00)	3.00 (2.00–4.00)	2.00 (2.00–3.00)	0.00 (0.00–0.00)	0.00 (0.00–0.75)

* IQR, interquartile range; IVIG, intravenous immunoglobulin; LVV, large vessel vasculitis; MAGIC, mouth and genital ulcers and inflamed cartilage; PGA, physician global assessment; SMVV, small/medium vessel vasculitis; VEXAS, vacuoles, E1 enzyme, X‐linked, autoinflammatory, somatic syndrome.

#### 2. Performance characteristics of anti‐Col2 antibodies and levels across diseases

**Figure 1:**
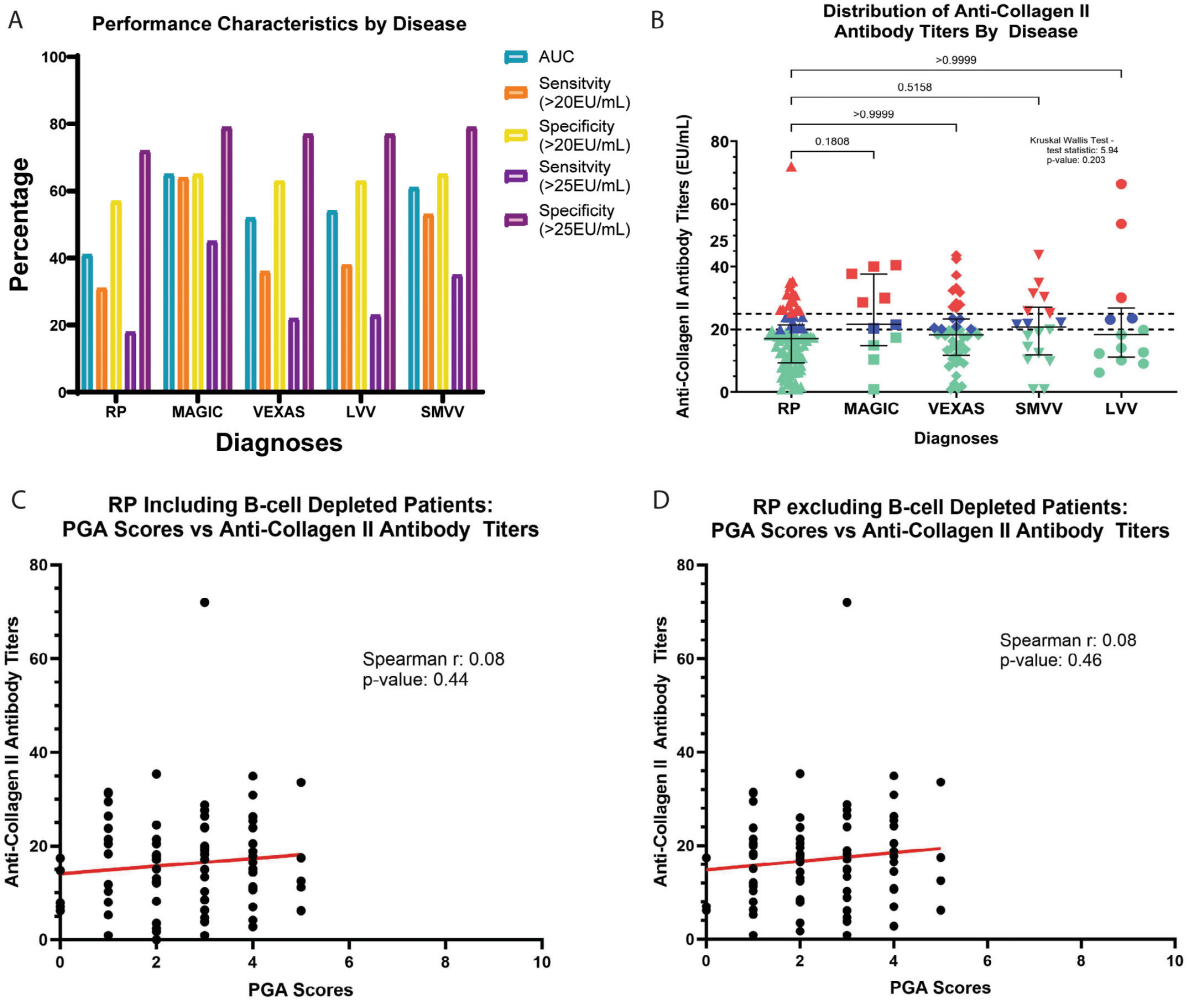
Anti‐Col2 antibodies and clinical associations. (A) Prevalence of anti‐Col2 antibodies at two different titers did not differ between RP, MAGIC syndrome, VEXAS, LVV, and SMVV. (B) Anti‐Col2 antibodies did not differ by disease where green indicates normal, blue is borderline elevated, and red is elevated. (C) Anti‐Col2 antibody titers were not associated with PGA scores of disease activity in patients with RP (D) or when excluding patients with RP who were B cell depleted. AUC, area under the curve; Col2, collagen II; EU, equivalent unit; LVV, large vessel vasculitis; MAGIC, mouth and genital ulcers with inflammatory chondritis; PGA, physician global assessment; RP, relapsing polychondritis; SMVV, small/medium vessel vasculitis; VEXAS, E1 enzyme, X‐linked, autoinflammatory, somatic.

The performance characteristics of anti‐Col2 antibodies to diagnose RP were poor at a reference cutoff of both >20 EU/mL (sensitivity = 31%; specificity = 57%) and of >25 EU/mL (sensitivity = 18%; specificity = 72%). The prevalence of anti‐Col2 antibodies was greater in the comparator diseases (28%) than RP (18%), with an area under the curve <50% observed for RP relative to comparators (Figure 1A; Supplementary Figure 1). There were no significant differences in the proportion of patients with detectable anti‐Col2 antibodies by disease (RP = 15 of 84, 18%; MAGIC = 5 of 11, 46%; LVV = 3 of 13, 23%; SVV = 6 of 18, 33%; VEXAS = 10 of 45, 22%; *P* = 0.21). The performance characteristics of anti‐Col2 antibodies for each disease was poor (Figure 1A). No significant differences in median anti‐Col2 antibody titer levels were observed between diseases (RP = 17.1 EU/mL; MAGIC = 21.6 EU/mL; VEXAS = 18.2 EU/mL; LVV = 18.3 EU/mL; SMVV = 20.8 EU/mL; *P* = 0.20, Figure 1B).

#### 3. Correlation of anti‐Col2 antibody levels and disease activity

Anti‐Col2 antibody titers did not correlate with disease activity for any of the diseases (RP *r* = 0.08, *P* = 0.44; MAGIC *r* = 0.01, *P* = 0.99; VEXAS *r* = −0.05, *P* = 0.76; LVV *r* = 0.03, *P* = 0.94; SMVV *r* = 0.10, *P* = 0.70). Results did not change when excluding CD19 B cell–depleted patients (RP *r* = 0.08, *P* = 0.46; MAGIC *r* = 0.29, *P* = 0.45; VEXAS *r* = −0.21, *P* = 0.46; LVV *r* = 0.04, *P* = 0.91; SMVV *r* = 0.05, *P* = 0.88). Anti‐Col2 titers did not correlate with PGA in patients with RP, even when excluding those who were B cell depleted (Figure 1C and 1D). Comparing anti‐Col2 positivity in patients with RP who were in remission or had low disease activity (defined as a PGA score of 0 or 1) to those with active disease (defined as PGA score ≥2), no statistically significant association was observed (21% vs 15%, *P* = 0.73). No associations were observed between anti‐Col2 antibody titer levels and specific comparator diseases (Supplementary Table 2).

#### 4. Anti‐Col2 antibody association with specific clinical manifestations of RP

At a reference cutoff of >25 EU/mL, anti‐Col2 antibody positivity did not correlate with symptoms of chondritis or cartilage damage in patients with RP (Supplementary Figure 2A). Across all studied clinical associations, anti‐Col2 antibodies were only detected in a greater proportion of patients with RP who had ear chondritis (93 vs 87%) or inflammatory arthritis (93 vs 86%), but these results were not statistically significant. In patients with diseases that have known cartilage involvement (RP, VEXAS, MAGIC, and GPA), there was no association between anti‐Col2 antibody positivity and symptoms of chondritis or cartilage damage (Supplementary Figure 2B).

### Systematic review and meta‐analysis of anti‐Col2 antibodies in RP and related diseases

#### 1. Study characteristics

**Figure 2:**
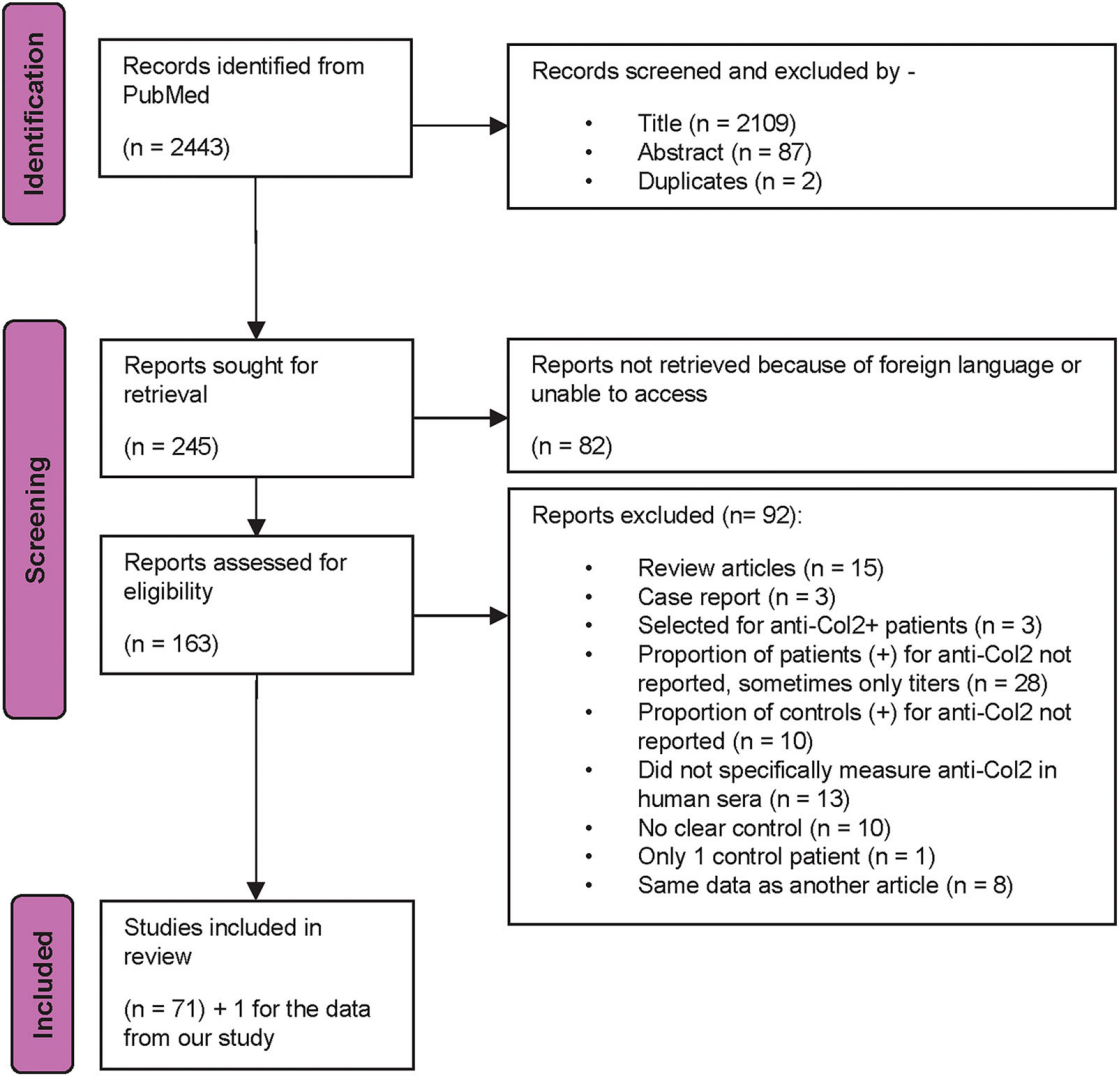
Systematic review study selection flow diagram. This flow diagram describes the process for identifying 71 articles included in this review out of 2,443 potential articles identified by the search strategy. Color figure can be viewed in the online issue, which is available at http://onlinelibrary.wiley.com/doi/10.1002/acr.25697/abstract.

Out of 2,443 articles screened, 71 met the inclusion criteria. Most exclusions were due to animal only studies, missing control data, or inaccessible text. The PRISMA flow diagram is show in Figure 2.

#### 2. Anti‐Col2 antibodies in RP

**Figure 3:**
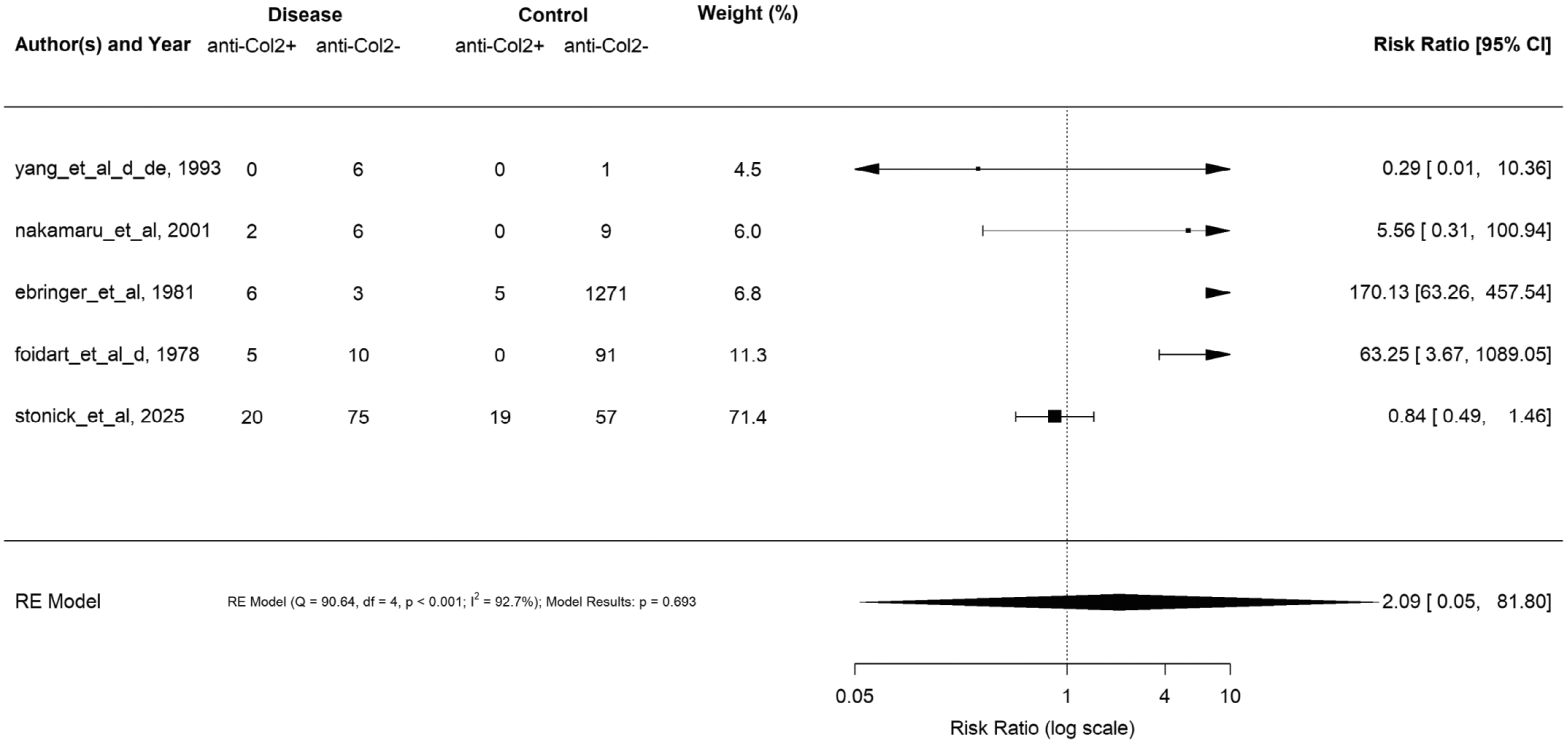
Forest plots of the association of anti‐Col2 antibodies and relapsing polychondritis. Anti‐Col2 antibodies were not associated with relapsing polychondritis across five pooled studies. The number of disease cases of relapsing polychondritis and controls is reported for each study along with individual and pooled risk ratios. CI, confidence interval; Col2, collagen II; RE, random effects.

Five studies, including the present observational cohort, assessed anti‐Col2 antibody positivity prevalence in RP compared to controls, and these studies were conducted between 1978 and 2025.^4^, ^5^, ^16^, ^17^ The pooled RR for these studies was 2.09 (95% CI: 0.05–81.80; *P* = 0.69) (Figure 3). The quality of articles ranged from 4 to 7 of 9 with a mean of 5.2 ± 1.3 (95% CI: 3.6–6.8). When studies were excluded based on a low‐quality score (≤4 of 9), the pooled RR decreased to 1.66 (95% CI: 0.07–41.30; *P* = 0.76) (Supplementary Figure 3). There was significant heterogeneity between the studies (*I* ^2^ = 92.7%; *P* < 0.001).

#### 3. Anti‐Col2 antibodies in other diseases

**Figure 4:**
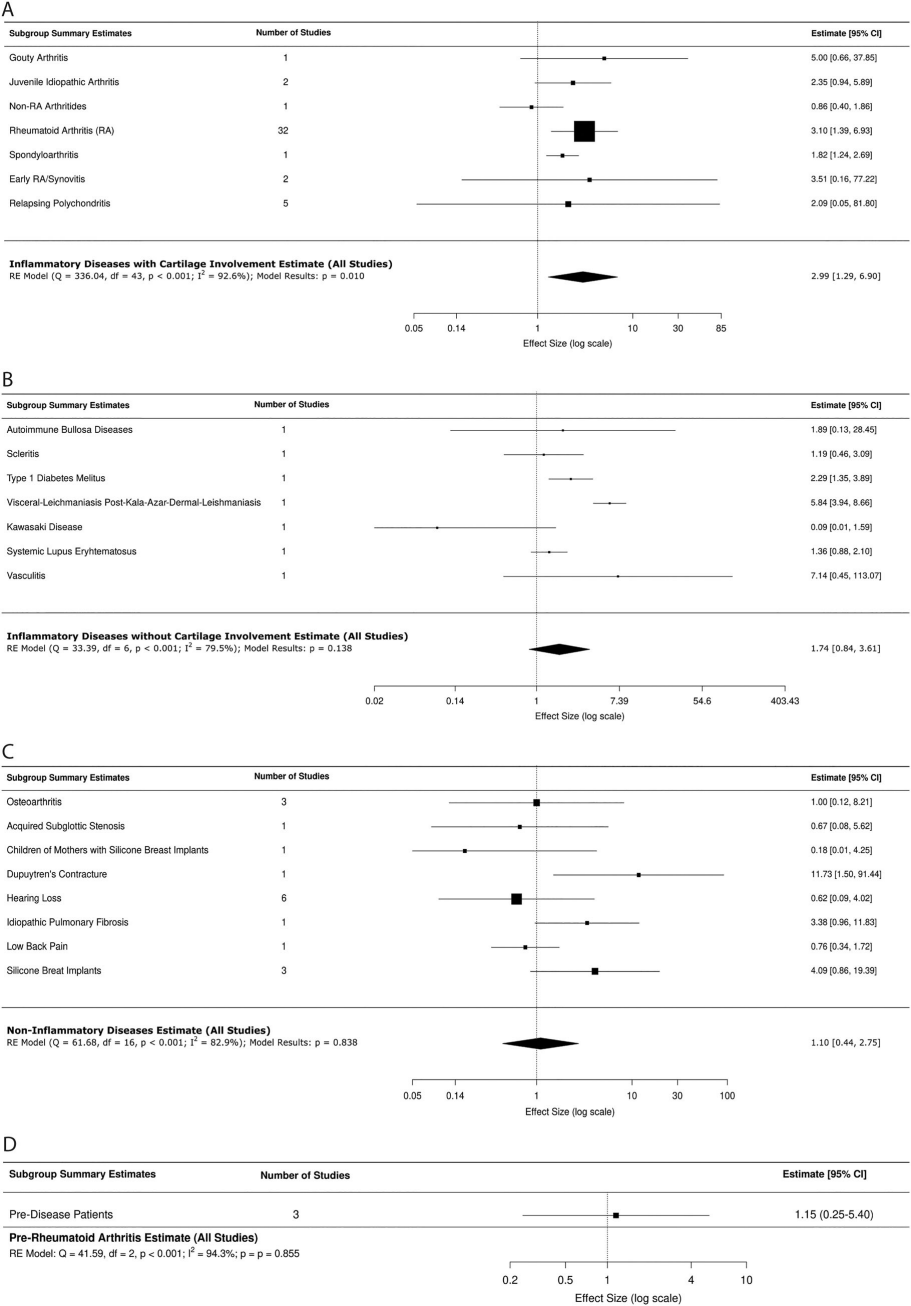
Forest plots of the association of anti‐Col2 antibodies and a broad spectrum of clinical diseases. (A) Pooled risk ratios show a significant association between anti‐Col2 antibodies and inflammatory diseases with cartilaginous involvement. Significant associations were not detected between (B) inflammatory diseases without cartilaginous involvement, (C) noninflammatory diseases, (D) or in studies in which antibody testing was performed before the onset of rheumatoid arthritis and anti‐Col2 anitbody positivity. CI, confidence interval; Col2, collagen II; RA, rheumatoid arthritis; RE, random effects.

There were 44 studies published between 1978 and 2025 comparing the prevalence of anti‐Col2 antibodies in inflammatory diseases with cartilaginous involvement to controls, including the present observational cohort study.^4^, ^5^, ^16^, ^17^, ^18^, ^19^, ^20^, ^21^, ^22^, ^23^, ^24^, ^25^, ^26^, ^27^, ^28^, ^29^, ^30^, ^31^, ^32^, ^33^, ^34^, ^35^, ^36^, ^37^, ^38^, ^39^, ^40^, ^41^, ^42^, ^43^, ^44^, ^45^, ^46^, ^47^, ^48^, ^49^, ^50^, ^51^, ^52^, ^53^, ^54^, ^55^, ^56^, ^57^ There was a significant association between anti‐Col2 antibodies and inflammatory diseases with cartilaginous involvement compared to controls (RR: 2.99; 95% CI: 1.29–6.91; *P* = 0.01) (Figure 4A). Because studies were weighted based on disease cohort size, statistical significance was largely driven by three larger studies—Too et al, which was weighted at 13.5%; Manivel et al, which was weighted at 16.2%; and Zeng et al, which was weighted at 23.2% (Supplementary Figure 4).^41^, ^53^, ^55^ There was a significant amount of heterogeneity between studies in this group (*I* ^2^ = 92.6%; *P* < 0.001). The Egger's test for study bias did not find significant study bias (*P* = 0.057). The line generated from Egger's test on the funnel plot had a negative slope, indicating a nonstatistically significant trend toward a bias in which smaller studies showed larger effect sizes (Supplementary Figure 5A).

There were seven studies published between 1988 and 2016 comparing the prevalence of anti‐Col2 antibodies in inflammatory diseases without cartilaginous involvement to controls.^58^, ^59^, ^60^, ^61^, ^62^, ^63^ Risk of anti‐Col2 antibodies in inflammatory diseases without cartilaginous involvement was not significantly different compared to controls (RR: 1.74, 95% CI: 0.84–3.61; *P* = 0.11) (Figure 4B, Supplementary Figure 6). There was a significant amount of heterogeneity between the studies within this group (*I* ^2^ = 79.5%; *P* < 0.001). The Egger's test for study bias did not find significant study bias (*P* = 0.13) (Supplementary Figure 5B). The line generated from Egger's test on the funnel plot had a positive slope indicating bias toward larger studies showing larger effect sizes.

There were 17 studies published between 1979 and 2018 examining the prevalence of anti‐Col2 antibodies in noninflammatory diseases,^64^, ^65^, ^66^, ^67^, ^68^, ^69^, ^70^, ^71^, ^72^, ^73^, ^74^, ^75^, ^76^, ^77^, ^78^, ^79^ and three studies published between 1988 and 2015 focused on anti‐Col2 antibodies in a predisease state before onset of RA.^80^, ^81^, ^82^ Compared to controls, there was no significant association between the noninflammatory group (RR: 1.10, 95% CI: 0.44–2.75; *P* = 0.84) or the predisease group (RR: 1.15, 95% CI: 0.25–5.40; *P* = 0.86) (Figure 4C and 4D; Supplementary Figures 7 and 8). There was a significant amount of heterogeneity between the studies within these groups (*I* ^2^ = 82.9%; *P* < 0.001 and *I* ^2^ = 94.3%; *P* < 0.001 respectively). The Egger's test for the noninflammatory disease category did not find significant study bias (*P* = 0.40). The line generated from Egger's test on the funnel plot had a negative slope indicating bias toward smaller studies showing larger effect sizes (Supplementary Figure 5C). The Egger's test for the predisease category did not find significant study bias (*P* = 0.83). The line generated from Egger's test on the funnel plot had positive slope indicating bias toward larger studies showing larger effect sizes (Supplementary Figure 5D).

#### 4. Meta‐regressions

**Figure 5:**
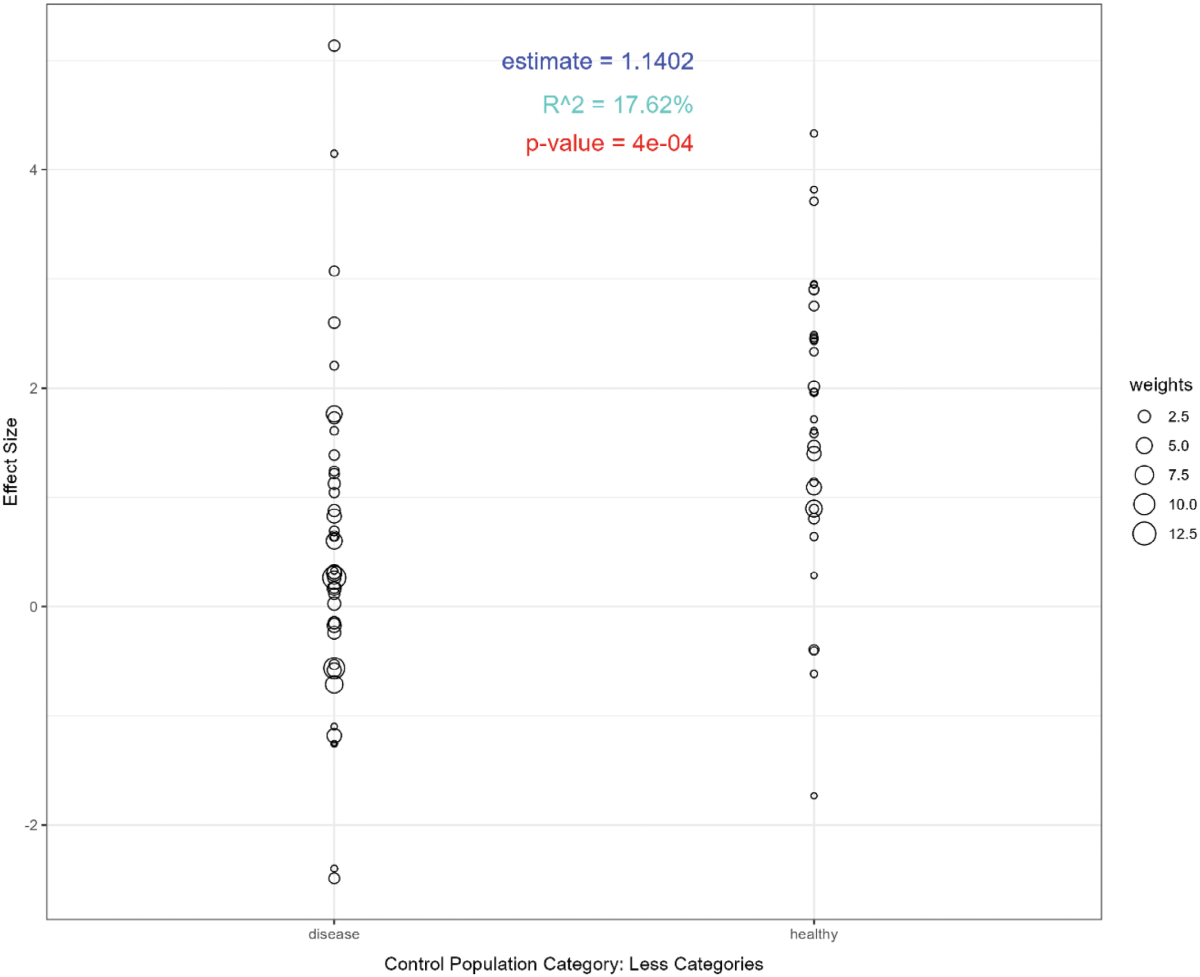
Meta‐regression to determine sources of heterogeneity between studies. Studies compared the prevalence of anti‐Col2 antibodies using healthy controls rather than disease controls reported significantly higher effect estimates. Col2, collagen II. Color figure can be viewed in the online issue, which is available at http://onlinelibrary.wiley.com/doi/10.1002/acr.25697/abstract.

On univariable regression, only one variable was significantly associated with interstudy heterogeneity with an *R* ^2^ value >0%. Use of healthy controls rather than disease comparators was significantly associated with an increased effect size (β estimate = 1.14, *P* = 0.0004; Figure 5). However, this variable only explained 17.08% of the heterogeneity.

## DISCUSSION

Anti‐Col2 antibodies have poor performance characteristics to diagnose or monitor disease activity in RP. Within the cohort analysis portion of this study, the presence of anti‐Col2 antibodies in patients with RP was not associated with diagnosis, disease activity, or any specific clinical characteristics of disease. Depending on the titer level used to define anti‐Col2 antibody positivity, only 18% to 32% of patients with RP had detectable antibodies. There was a numerically greater proportion of patients with RP who had detectable anti‐Col2 antibodies in association with ear chondritis and inflammatory arthritis—which are phenotypic features that mice sensitized with anti‐Col2 antibodies can develop.^8^, ^9^ However, these associations were not statistically significant and were not replicated in a broader set of diseases defined by cartilage involvement. More broadly, the systematic review and meta‐analysis portion of the study confirmed that beyond a lack of sensitivity, anti‐Col2 antibodies are also not specific for RP and can be detected at similar frequencies across a wide range of related and unrelated conditions. Therefore, anti‐Col2 antibodies are not useful in clinical practice to diagnose a patient with suspected RP or to monitor disease activity in a patient with confirmed RP.

Anti‐Col2 antibodies have been studied across a wide spectrum of diseases. Although there was significant variability in individual study results within the meta‐analysis, a significant pattern of association was observed. Compared to noninflammatory diseases, there was an association between the presence of anti‐Col2 antibodies and inflammation, with a three‐fold increased risk to detect anti‐Col2 antibodies in a group of diseases defined by inflammation with cartilage involvement that was statistically significant and a near significant two‐fold increased risk in a group of inflammatory diseases without cartilage involvement. For the few studies in which anti‐Col2 antibodies were measured in the predisease state, there was no clinical association. Taken together, these findings suggest that anti‐Col2 antibodies may form in response to systemic inflammation that involves cartilage or collagen rich structures but are less likely to play a primary role in disease pathogenesis for a specific condition.

Significant heterogeneity was observed between the studies included in this meta‐analysis. Meta‐regression did not identify factors that accounted for a substantial proportion of heterogeneity between studies. The only variable that was significantly associated with heterogeneity was the composition of the control group. Studies that used disease controls rather than healthy controls were significantly less likely to identify an association between anti‐Col2 antibodies and the disease of interest, supporting the concept that anti‐Col2 antibodies are not disease specific and can be seen in a range of other conditions.

The current cohort study has several strengths. First, compared to prior studies, this is the largest cohort of patients with RP for whom anti‐Col2 antibodies were tested. Second, the cohort study compared RP to a spectrum of related diseases rather than healthy controls. Choosing an appropriate comparator population is crucial because the clinical utility of any diagnostic test is only as good as its ability to differentiate diseases with similar presentations. Third, this study contains the only systematic review and meta‐analysis performed to date to determine the clinical utility of anti‐Col2 antibodies as a diagnostic biomarker. The study was performed in accordance with PRISMA recommendations. Results from the meta‐analysis aligned with the cohort study to provide robust cumulative evidence about the poor performance characteristics of anti‐Col2 antibodies to diagnose RP due to low sensitivity and specificity. Fourth, we detected a novel association between anti‐Col2 antibodies and cartilage inflammation that was not disease‐specific and could inform future experiments regarding the role of anti‐Col2 antibodies in the context of cartilage inflammation.

This study also has potential limitations to consider. In the cohort study, anti‐Col2 antibody levels were typically measured later into disease in patients who were receiving immunosuppressive therapies. Anti‐Col2 antibodies may be higher earlier on in disease onset, such as in juvenile idiopathic arthritis.^21^ However, the performance characteristics of anti‐Col2 antibodies were poor even when excluding patients on B cell–depleting therapies, and no association was seen between anti‐Col2 and disease activity. Disease activity scores differed at the time of antibody assessment, with the highest scores observed in patients with RP, MAGIC, and VEXAS. Despite these differences, an increased prevalence of anti‐Col2 antibodies was not observed in these conditions relative to other disease comparators. The Mayo Clinic (test identifier: FFTYC) Collagen II Antibodies test recognizes a specific epitope, and it is possible that another epitope would show a more significant correlation. Nevertheless, this is the major commercially available test for anti‐Col2 antibodies, and thus it is a reasonable epitope on which to analyze performance characteristics. Additionally, the studies analyzed in the systematic review and meta‐analysis used a wide range of different antigens and epitopes, yet no consistent pattern of anti‐Col2 antibody positivity was associated with a particular disease or cartilage antigen. For the systematic review and meta‐analysis, diseases were categorized based on study investigator agreement, so there was potential for misclassification, particularly when differentiating between inflammatory diseases with or without cartilage involvement. The number of articles included within each disease category was variable, and few articles were identified in the predisease group, which may limit the precision of the findings and introduce bias. The definitions for normal varied across the studies, so it can be challenging to know precisely how the deviations in the definitions may have affected positivity rates in the individual study cohorts. However, meta‐regression analysis based on study definitions of normal did not reveal a significant contribution to interstudy heterogeneity.

In conclusion, anti‐Col2 antibodies have limited utility in clinical practice. These antibodies are neither sensitive nor specific to diagnose RP, and they do not correlate with disease activity or specific clinical features. Anti‐Col2 antibodies may be indiscriminately detected in a wide range of diseases in which there is inflammation of cartilage, indicating that they lack the specificity required for them to be used for clinical diagnosis or disease monitoring. Despite a lack of clinical utility, there is continued rationale to study the development and function of anti‐Col2 antibodies in research settings. Identification of a diagnostic biomarker that can differentiate RP from related conditions remains a research priority and a major unmet clinical need.

## AUTHOR CONTRIBUTIONS

All authors contributed to at least one of the following manuscript preparation roles: conceptualization AND/OR methodology, software, investigation, formal analysis, data curation, visualization, and validation AND drafting or reviewing/editing the final draft. As corresponding author, Dr Grayson confirms that all authors have provided the final approval of the version to be published and takes responsibility for the affirmations regarding article submission (eg, not under consideration by another journal), the integrity of the data presented, and the statements regarding compliance with institutional review board/Declaration of Helsinki requirements.

## Supporting information


**Disclosure form**.

**Appendix S1:** Supplementary Information.
