# Supplementary material for: Performance Characteristics of Anti–Collagen II Antibodies in Relapsing Polychondritis and Related Diseases: Prospective Analysis, Systematic Review, and Meta‐Analysis
Source: Arthritis Care Res (Hoboken). 2026 Feb 11;78(6):798–809. doi: 10.1002/acr.25697 (PMC13206174; doi:10.1002/acr.25697)
Supplement: Supplementary file 2 — Appendix S1: Supplementary Information. [file ACR-78-798-s002.docx]

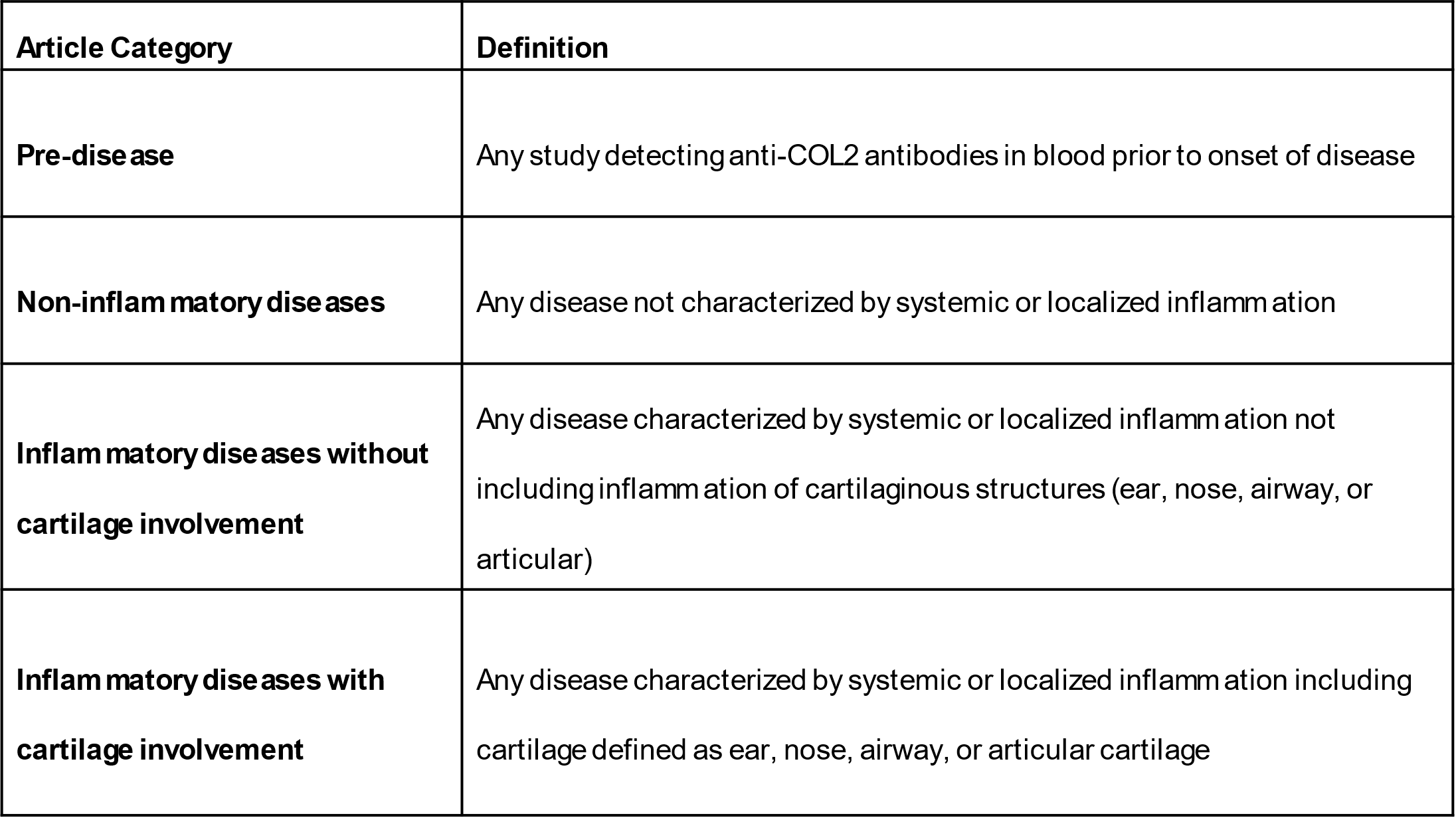


**Supplementary Table 1: Definitions for study characterization.** Anti-Col2 = anti-collagen II.


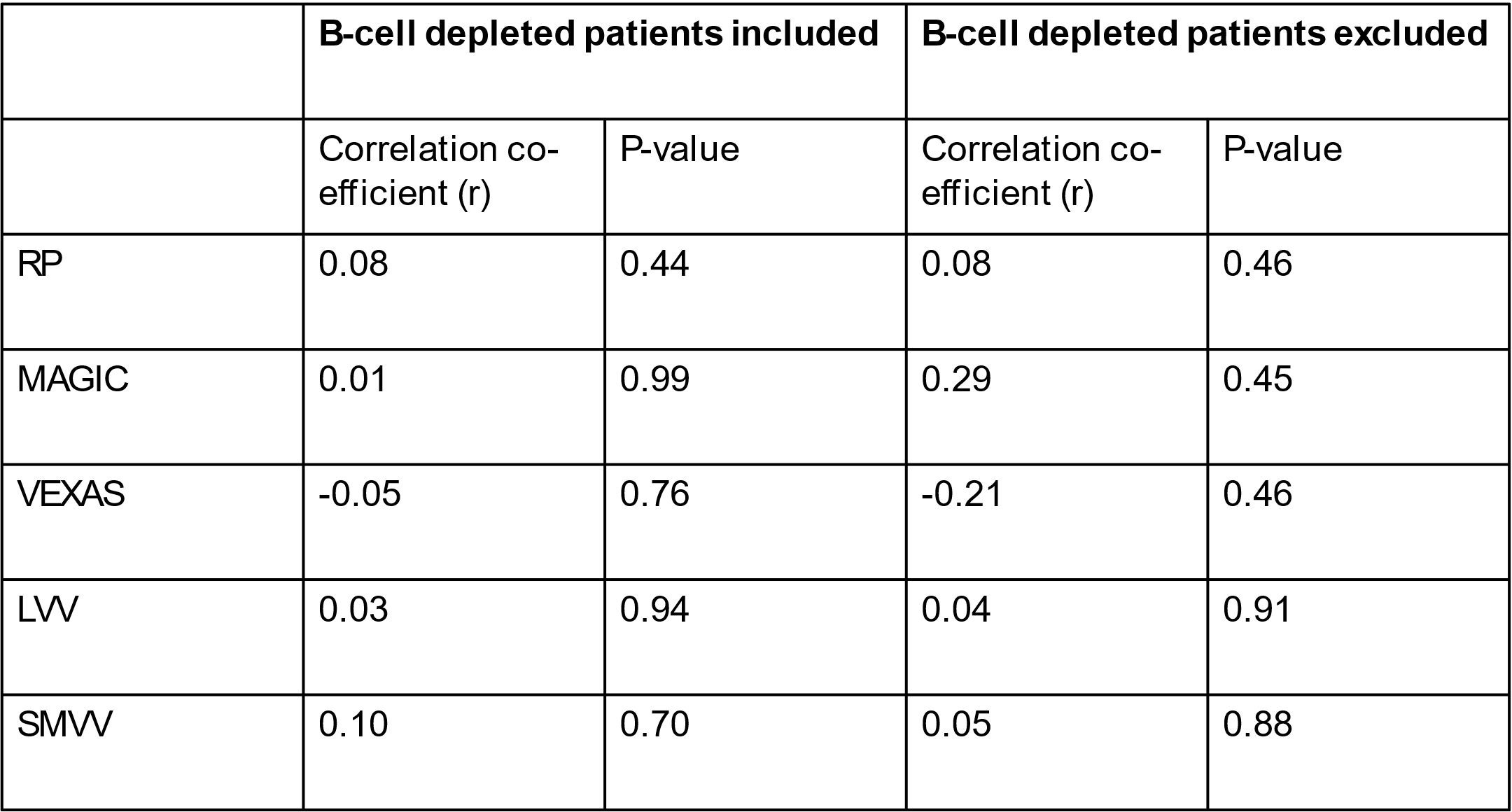


**Supplementary Table 2: Correlation between disease activity and anti-collagen II (anti-Col2) titer levels.** Disease activity was evaluated using physician global assessment (PGA) scores which range from 0 – no disease activity to 10 – severe disease activity.


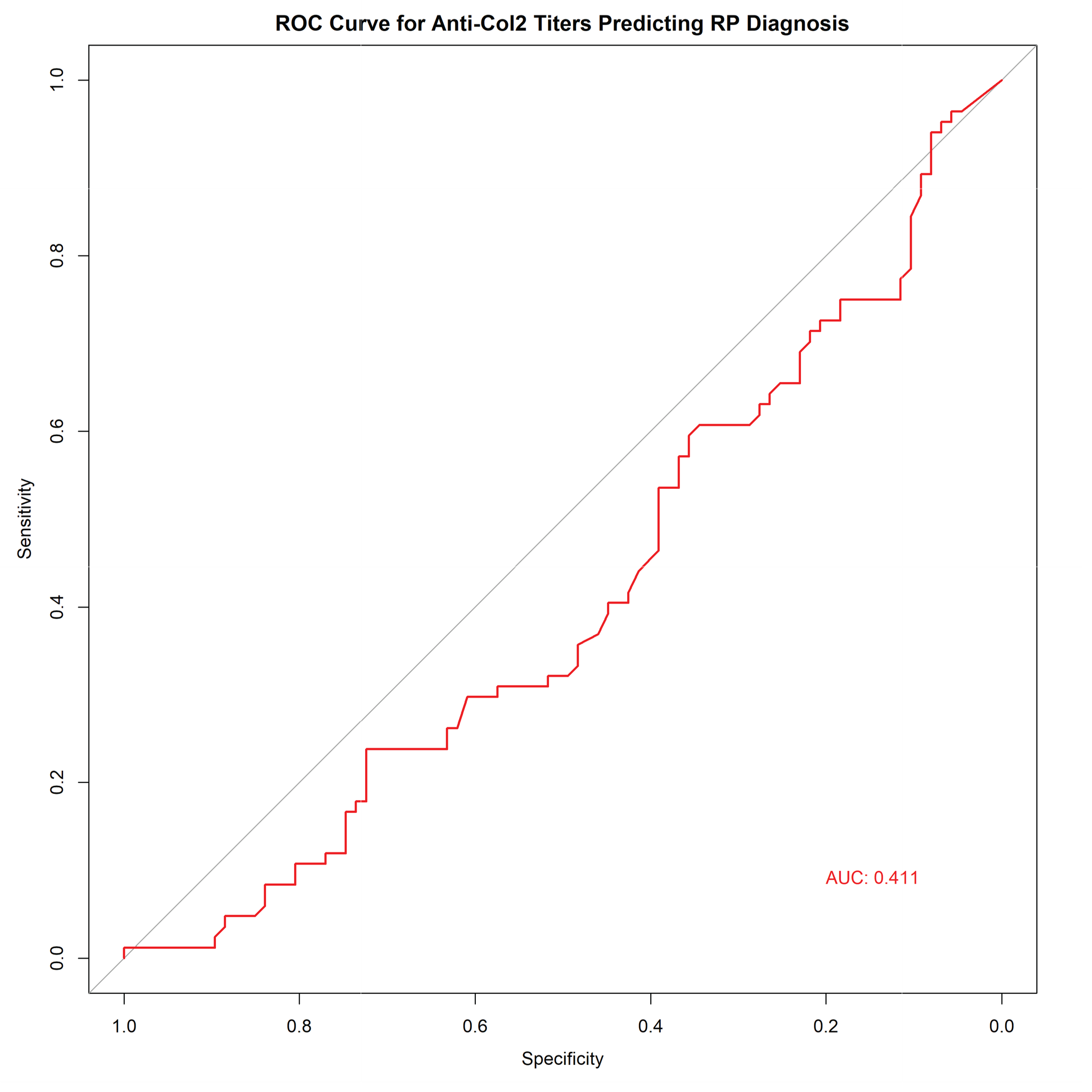


**Supplementary Figure 1: Receiver operating characteristic (ROC) curve for anti-collagen II (anti-Col2) antibody’s ability to diagnosis relapsing polychondritis (RP).** For this ROC curve, the parameters were set so that control = 0 and RP = 1.

A

B

**Supplementary Figure 2: Anti-collagen II (anti-Col2) antibody positivity in association with chondritis and cartilage damage.** The association of clinical features of chondritis and cartilage damage with anti-Col2 antibody positivity (>25 EU/mL) was evaluated in (A) relapsing polychondritis (RP) and in (B) diseases with cartilaginous involvement (RP, VEXAS, MAGIC, and GPA). VEXAS = vacuoles, E1 enzyme, X-linked, autoinflammatory, somatic syndrome; MAGIC = mouth and genital ulcers and inflamed cartilage; GPA = granulomatosis with polyangiitis; SGS = subglottic stenosis; TM = tracheomalacia; BM = bronchomalacia.


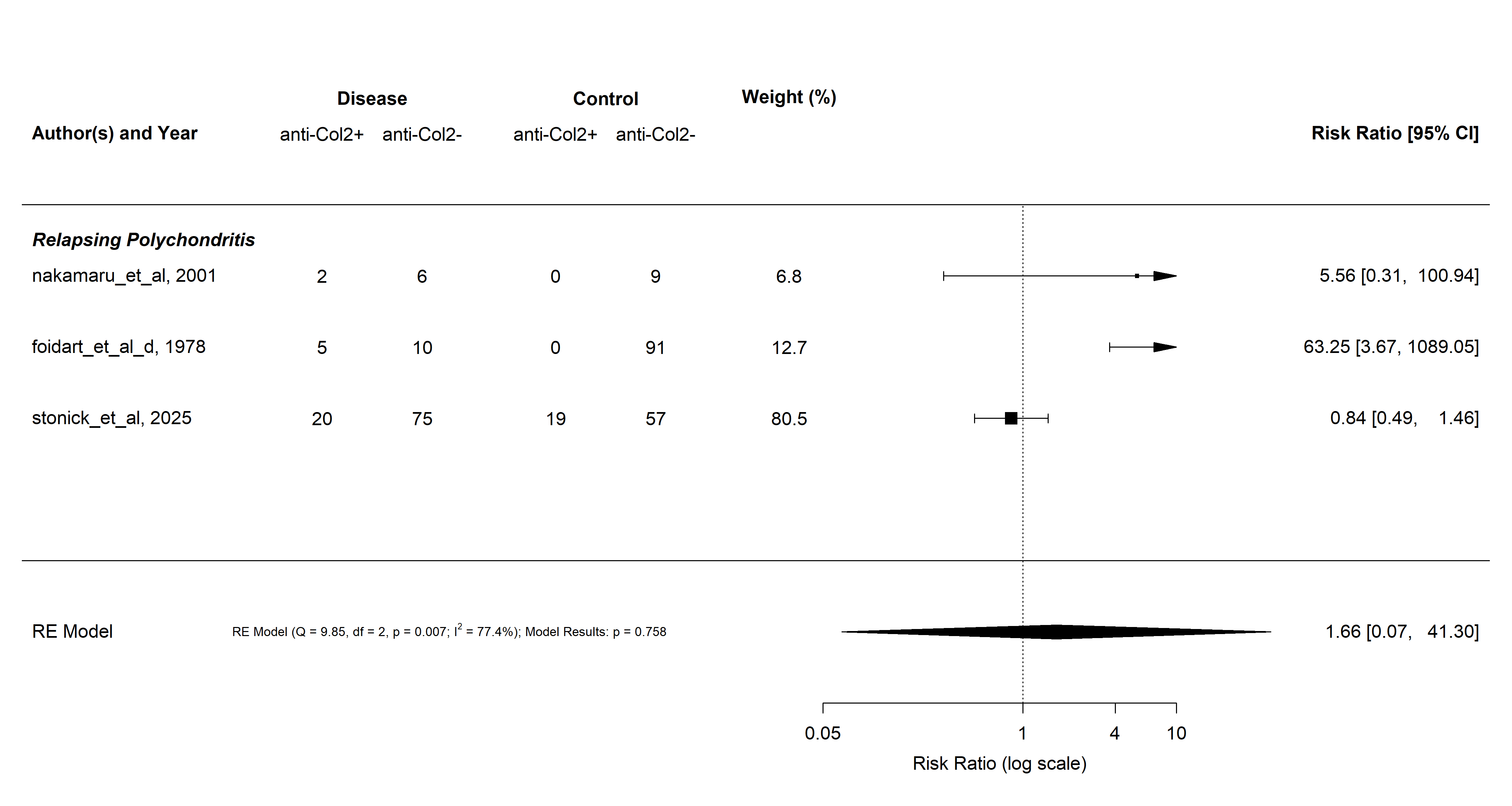


**Supplementary Figure 3: Forest plots of the association of anti-collagen II (anti-Col2) antibodies and relapsing polychondritis** **excluding low quality articles.** Low quality articles were defined as those with a quality score of 4/9 and below. CI = confidence internal; RE = random effects.


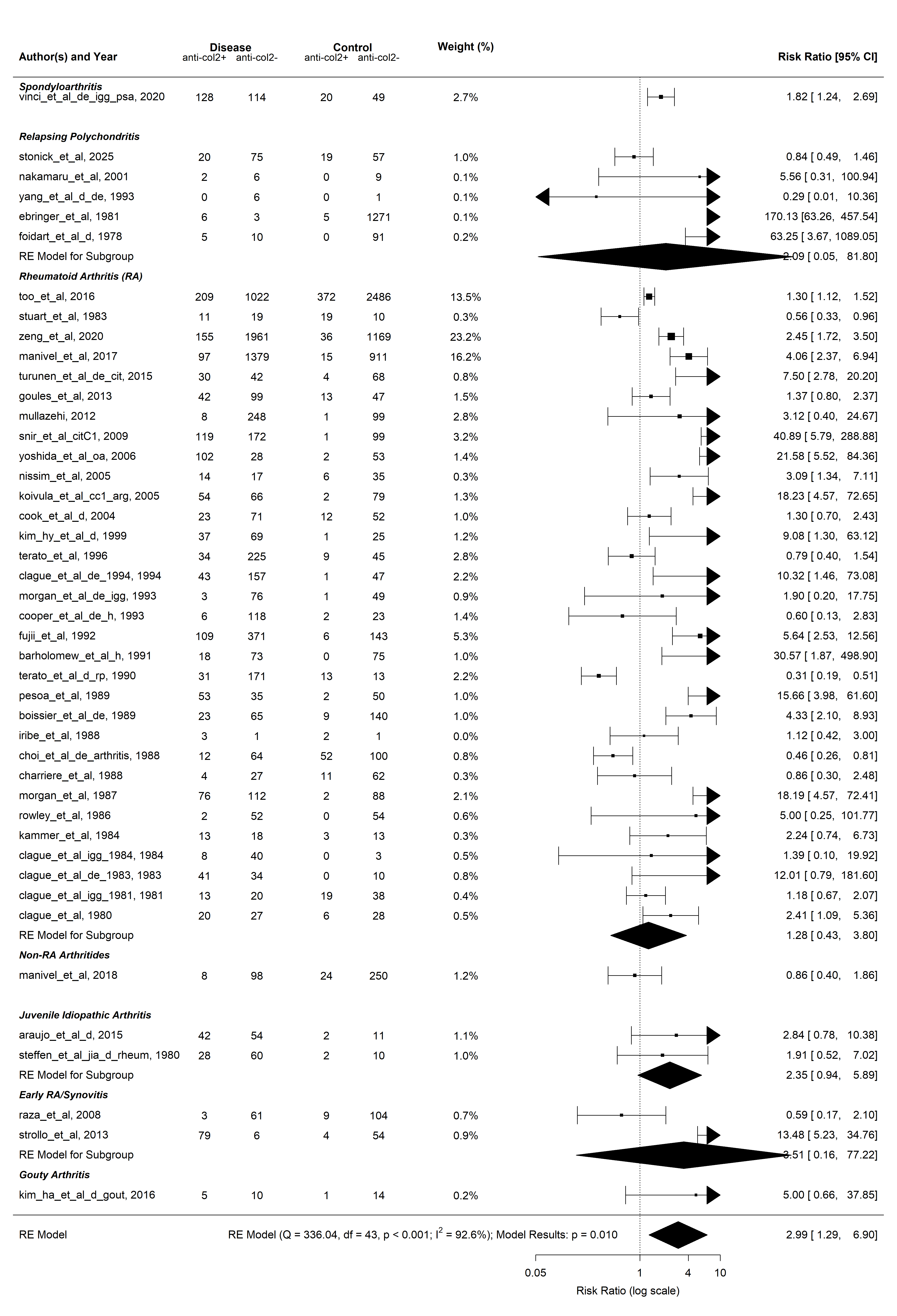


**Supplementary Figure 4: Forest plots for inflammatory diseases with cartilage involvement.** Risk ratios (RRs) for each individual study in this disease category are shown as well as the pooled category and disease RRs. CI = confidence interval; RE = random effects.


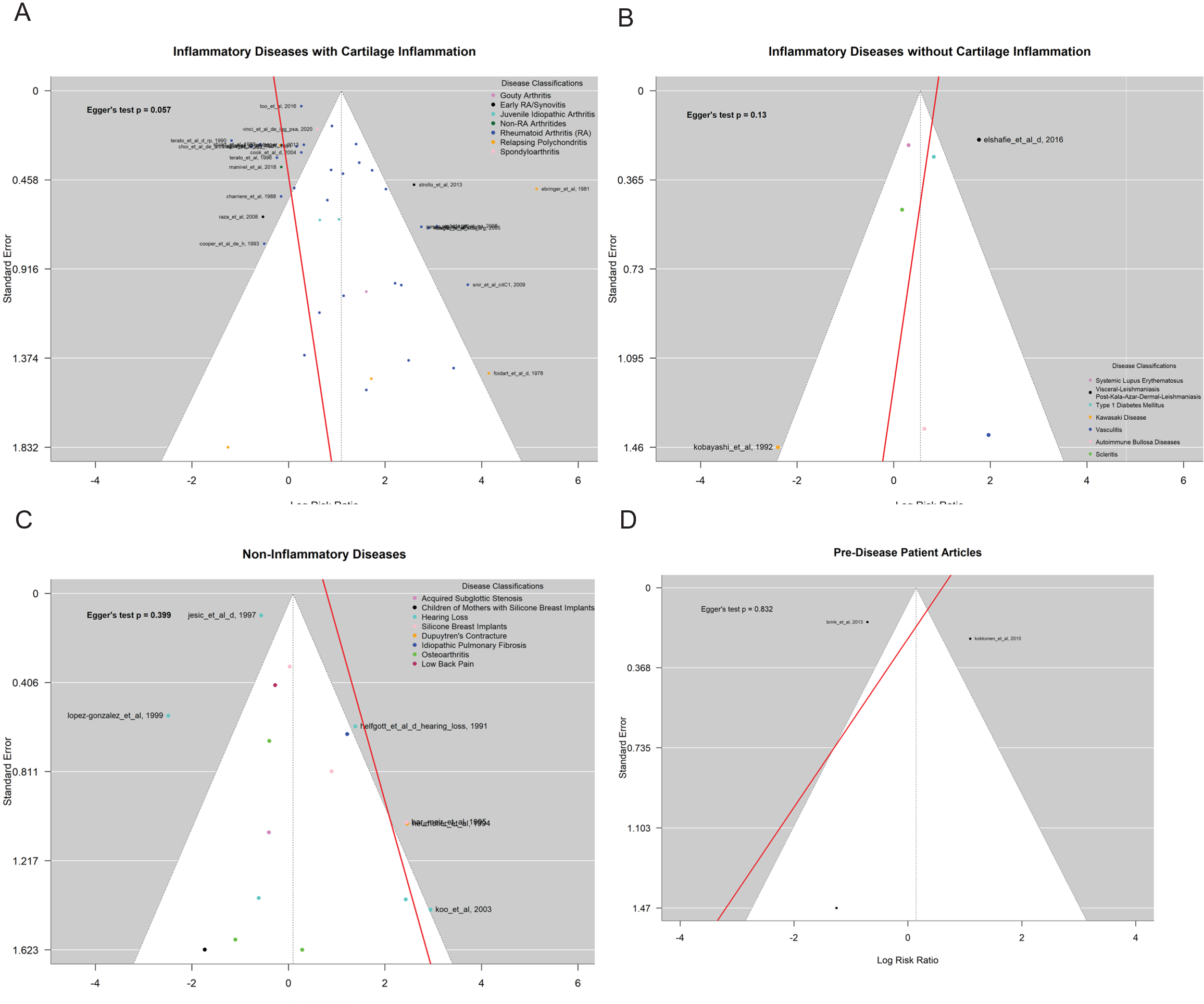


**Supplementary Figure 5: Funnel plots to examine potential bias.** Significant bias was not observed for studies where the disease cohort includes inflammatory diseases with cartilaginous involvement (A), inflammatory diseases without cartilaginous involvement (B), non-inflammatory diseases (C), or patients tested prior to development of rheumatoid arthritis (D).


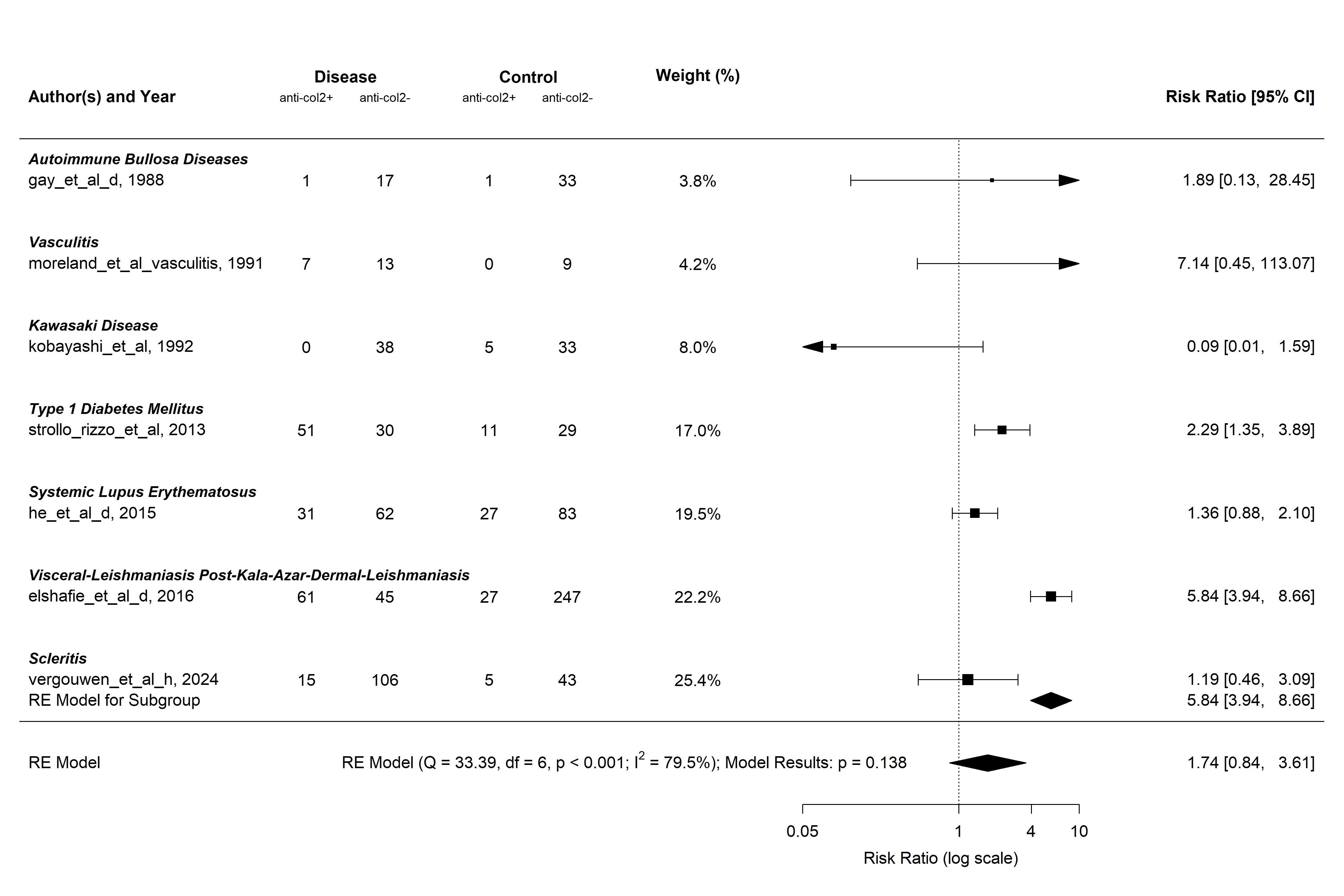


**Supplementary Figure 6: Forest plots for inflammatory diseases without cartilage involvement.** Risk ratios (RRs) for each individual study in this disease category are shown as well as the pooled category and disease RRs. CI = confidence interval; RE = random effects.

**
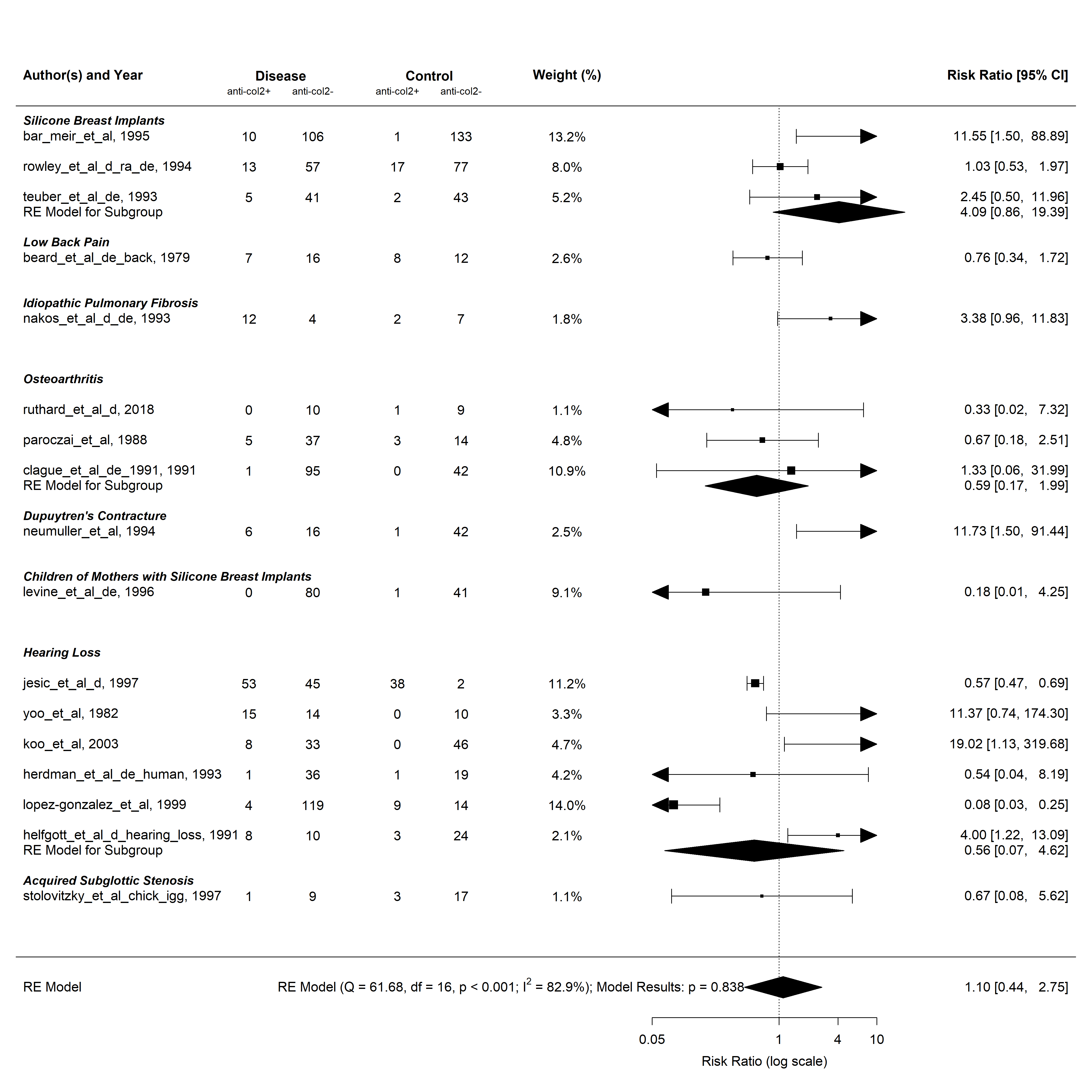
**

**Supplementary Figure 7: Forest plots for noninflammatory diseases.** Risk ratios (RRs) for each individual study in this disease category are shown as well as the pooled category and disease RRs. CI = confidence interval; RE = random effects.


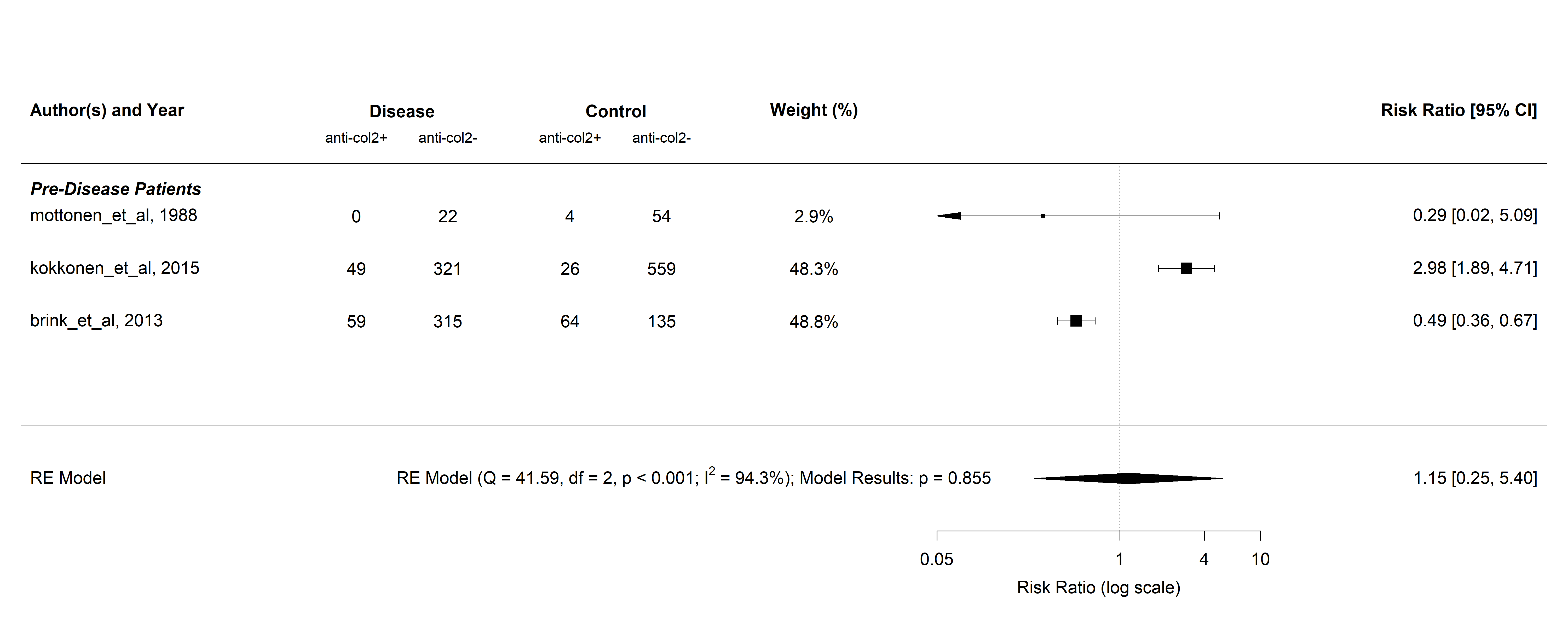


**Supplementary Figure 8: Forest plots for studies evaluating pre-disease patients.** Risk ratios (RRs) for each individual study in this disease category are shown as well as the pooled category RR. All pre-disease patients were individuals who later developed rheumatoid arthritis (RA). CI = confidence interval; RE = random effects.
